# The Efficacy and Adverse Events in Patients with Head and Neck Cancer Following Radiotherapy Combined with S-1 Therapy: A Meta-Analysis

**DOI:** 10.3390/cancers13122971

**Published:** 2021-06-13

**Authors:** Hung-Sheng Shih, Hong-Jie Jhou, Yang-Hao Ou, Yen-Tze Liu, Chew-Teng Kor, Andy Wei-Ge Chen, Mu-Kuan Chen

**Affiliations:** 1 Division of General Practice, Department of Medical Education, Changhua Christian Hospital, Changhua 500, Taiwan; 182949@cch.org.tw or; 2 Department of Neurology, Changhua Christian Hospital, Changhua 500, Taiwan or xsai4295@gmail.com (H.-J.J.); or yanghao.ou@gmail.com (Y.-H.O.); 3 Institute of Medicine, Chung Shan Medical University, Taichung 402, Taiwan; 144084@cch.org.tw; 4 Department of Family Medicine, Changhua Christian Hospital, Changhua 500, Taiwan; 5 Department of Holistic Wellness, Mingdao University, Changhua 500, Taiwan; 6 Oral Cancer Research Center, Changhua Christian Hospital, Changhua 500, Taiwan; 7 Big Data Center, Changhua Christian Hospital, Changhua 500, Taiwan; 179297@cch.org.tw; 8 Graduate Institute of Statistics and Information Science, National Changhua University of Education, Changhua 500, Taiwan; 9 Department of Otolaryngology-Head & Neck Surgery, Changhua Christian Hospital, Changhua 500, Taiwan

**Keywords:** head and neck cancer, metronomic chemotherapy, radiotherapy

## Abstract

**Simple Summary:**

Head and neck squamous cell carcinoma (HNSCC) ranks sixth among the most common cancers, accounting for approximately 3.3% of all cancer cases. While HNSCC was generally treated with postoperative concurrent chemo-radiotherapy, traditional chemotherapy agents often lead to multiple side effects. On the other hand, continuous loss dose oral metronomic chemotherapy, such as S-1, maintains good anticancer activity and involves fewer side effects. While several studies have been undertaken on metronomic chemotherapy in head and neck cancer (HNC) during the last decade, results have often been contradictory. We addressed the contradictory literature with a meta-analysis to summarize the available evidence on the efficacy of S-1 chemotherapy combined with radiotherapy for patients with head and neck cancer and the adverse effects associated with the therapy.

**Abstract:**

This meta-analysis was conducted to assess the efficacy and adverse events associated with S-1 chemotherapy combined with radiotherapy for patients with head and neck cancer. The PubMed, Embase, and Cochrane Library databases were searched up to 10 February 2021. Eligible studies included clinical trials using S-1 chemotherapy combined with radiotherapy for head and neck cancer patients that measured tumor response, local control rate, overall survival, and grade 3/4 adverse events. A meta-analysis was performed using a random effects model. Twelve trials involving 378 patients met the selection criteria. The objective response and clinical benefit rate (complete/partial response and stable disease) of S-1 chemotherapy with radiotherapy were 86.3% (95% confidence interval (CI), 60.3–96.3) and 88.3% (95% CI, 70.1–96.1), respectively. The median 3-year local control rate, 3-year overall survival rate, and grade 3/4 adverse event rate were 84.0% (95% CI, 71.4–91.7), 69.6% (95% CI, 54.9–81.1), and 42.0% (95% CI, 36.2–48.0), respectively. S-1 combined with radiotherapy for patients with head and neck squamous cell carcinoma results in a good tumor response, favorable survival rate, and low toxicity. A prospective randomized, double-blind trial is required to assess the efficacy and safety of S-1 combined with radiotherapy to treat HNSCC.

## 1. Introduction

Head and neck squamous cell carcinoma (HNSCC) ranks sixth among the most common cancers, accounting for approximately 3.3% of all cancer cases [1]. The global incidence of head and neck cancer (HNC) is approximately 500,000 new cases each year, and the number of patients diagnosed with advanced cancer is increasing. Despite the continuous development of treatment strategies in recent years, the survival rate of HNC is still unsatisfactory.

Advanced stage HNSCC is often treated with postoperative concurrent chemo-radiation therapy (CRT) [2]. Traditional chemotherapy using the maximum tolerated dose usually produces serious side effects, and the patient often succumbs to treatment-resistant disease. In contrast, metronomic chemotherapy maintains good anticancer activity and involves less expensive chemotherapeutics [3,4]. Over the past decades, metronomic chemotherapy has been used in several different cancers, including breast cancer [3], lung cancer [5], and gastrointestinal cancer [6].

S-1, an oral fluoropyrimidine, is composed of tegafur, gimeracil (CDHP) and oteracil potassium. Gimeracil is a dihydropyrimidine dehydrogenase inhibitor that can maintain high concentrations of 5-fluorouracil (5-FU) [7]. An in vivo study undertaken on nude mice revealed that gimeracil can enhance radiosensitivity on oral squamous cell carcinoma cells [8]. Oteracil reduces the activity of 5-FU in the normal gastrointestinal mucosa and thus decreases gastrointestinal toxicity. Oteracil functions by blocking the enzyme orotate phosphoribosyltransferase (OPRT), which is involved in the production of 5-FU [9,10].

Several studies on the use of S-1 for HNSCC have been conducted over the past few decades. However, the results were often contradictory. The toxicity rate of S-1 for advanced or recurrent HNC differed from 46.2% to 28.8% in different phase II trials [11,12]. We addressed the contradictory literature with a meta-analysis to summarize the available evidence on the efficacy of S-1 chemotherapy combined with radiotherapy for patients with head and neck cancer and the adverse effects associated with the therapy.

## 2. Materials and Methods

We adhered to the Preferred Reporting Items for Systematic Reviews and Meta-Analyses (PRISMA) guidelines as well as a list within it (Table S1 PRISMA list) [13]. This review protocol is registered with the Open Science Framework platform (protocol available at https://osf.io/urmnh, last accessed on 11 June 2021).

### 2.1. Search Strategy

The following databases were searched up to 10 February 2021, for relevant studies: PubMed, Embase, and the Cochrane Library. Two independent investigators (Hung-Sheng Shih and Yang-Hao Ou) conducted a systematic search using the terms (“head and neck neoplasms” [MeSH] OR (“head” [All Fields] AND “neck” [All Fields] AND “neoplasms” [All Fields]) OR “head and neck neoplasms” [All Fields] OR (“head” [All Fields] AND “neck” [All Fields] AND “cancer” [All Fields]) OR “head and neck cancer” [All Fields]) AND “s-1” [All Fields]. Original articles, previous systematic reviews, and conference abstracts were manually screened to identify qualified studies. The search strategy has no language restrictions. If the data overlapped (e.g., data from the same clinical trial were included in two or more publications), the most complete and most recent report was selected for inclusion in the meta-analysis.

### 2.2. Study Selection

The following inclusion criteria were applied: (1) average patient age over 18 years, (2) observation and phase II or III prospective clinical trial of chemoradiotherapy (CRT) in patients with head and neck squamous cell carcinoma (HNSCC), (3) patients with normal liver, kidney and bone marrow function, and (4) sufficient data provided on tumor response, local-regional control rate (LCR), overall survival (OS) and adverse events (AEs). The exclusion criteria were as follows: (1) overlapping or duplicate publication; (2) studies in which necessary data could not be extracted; and (3) conference abstracts, reviews, letters, case reports.

### 2.3. Data Extraction and Outcome Measures

Two researchers (Hung-Sheng Shih and Yang-Hao Ou) independently extracted the relevant data and primary outcomes, including the 3-year OS (OS-3) rate, 3-year LCR (LCR-3), tumor response and grade 3/4 AEs. In case of disagreements, another author (Hong-Jie Jhou) was consulted, and decisions were made after a group discussion. The Response Evaluation Criteria in Solid Tumors (RECIST) were used to evaluate tumor response. CBR represents the proportion of patients who achieved complete remission (CR), partial remission (PR), or prolonged stable disease (pSD) ≥ 24 weeks; ORR represents patients who achieved CR or PR. Engauge Digitizer version 4.1 (Open-source software by Mark Mitchell, Torrance, CA, USA. Http://markummitchell.github.io/engauge-digitizer/, last accessed on 11 June 2021) was used to determine survival data by digitization if this information was not provided directly. Adverse events (AEs) were evaluated according to the National Cancer Institute Common Toxicity Criteria (NCICTC) and Common Terminology Criteria for Adverse Events (CTCAE). The sum of the different incidence rates of serious AEs was extracted from studies that recorded the composition of AEs. Other independent information recorded included the first author’s name, year of publication, country/region, study design, registration number, subject age, chemoradiotherapy schedule and number of evaluable patients.

### 2.4. Data Synthesis and Analysis

We investigated the data as recommended in the Cochrane Handbook for Systematic Reviews of Interventions [14]. A summary of the index is given in the form of incidence and has corresponding 95% confidence intervals (CIs). The I-squared (*I*^2^) statistic and Cochran’s Q test were used to assess heterogeneity. Statistically significant heterogeneity was defined as *I*^2^ > 50% and Cochran’s Q test *p* < 0.1, and a random effects model was used to summarize the heterogeneity effect size of each study [15]. Otherwise, a fixed effects model was chosen. We assessed the robustness of treatment effects on primary outcomes with a sensitivity analysis based on the 3-year overall survival rate, 3-year local control rate, objective response rate, and adverse events. A subgroup analysis of tumor localization was performed using Q statistics to detect tumor response, survival rate and statistical heterogeneity in the presence of observable heterogeneity. A *p*-value less than 0.05 (typically ≤ 0.05) was considered statistically significant. Meta-analysis was performed using Comprehensive Meta-Analysis Software System Software, Version 2 (Biostat, Englewood, NJ, USA).

### 2.5. Bias Assessment and Quality Assessment

Single-arm trials have a high risk of bias by nature; therefore, they were not further assessed for bias. Funnel plots were generated and evaluated with Egger tests to evaluate publication bias [16,17]. The quality of the observational comparative and non-comparative studies was appraised by Hung-Sheng Shih and Yang-Hao Ou using the Methodological Index for Non-Randomized Studies (MINORS) scale [18]. Any disagreement was resolved via group discussions [19]. The MINORS scale [20], which has been validated for both observational comparative and non-comparative studies, was introduced to evaluate the quality of the selected studies. Three articles in the meta-analyses were comparative studies with a control group, whereas the other nine studies were non-comparative. The study aim, patients, prospective data collection, assessment of the endpoint, follow-up duration, follow-up censor, and study size were collected for both non-comparative and comparative studies. An additional four items regarding the control group were applied to the comparative studies. The items were scored 0 (not reported), 1 (reported but inadequate), or 2 (reported and adequate). The global ideal score was 16 for non-comparative studies and 24 for comparative studies. We also used the Newcastle-Ottawa Scale (Table S2) [21]. The following aspects were evaluated: participant selection, study comparability, and outcome assessment.

## 3. Results

### 3.1. Search Results and Study Characteristics

**Figure 1:**
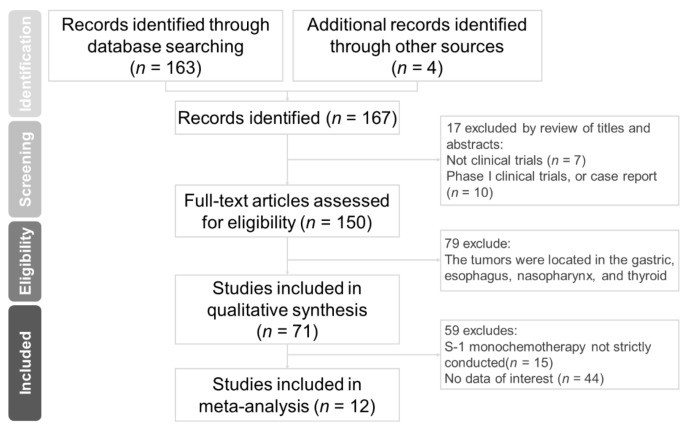
Flow diagram of the process used to select clinical trials.

The flow chart of the research is shown in Figure 1. A search of several electronic databases, including PubMed, Web of Science, Embase, and the Cochrane Library, yielded a total of 167 articles. Seventeen papers were excluded after a review of the titles and abstracts. Two of the most common reasons for exclusion were a lack of subject relevance (i.e., studies not related to concurrent S-1 chemotherapy and radiotherapy) and a non-clinical trial study design (Figure 1). Among the remaining 150 studies, 79 were further excluded after a review of the full-text. The reason for these exclusions was that the studies focused on cancers located in the esophagus, gastric tract, nasopharynx, and thyroid. Seventy-one potentially relevant articles were identified after we screened the full text. Interrelated data were not analyzed in 44 of these studies, and 15 trials did not strictly use S-1 as a monochemotherapy. Three randomized clinical trials and nine single-arm clinical trials with a total patient number of 378 were ultimately included in the present meta-analysis (Table 1) [22,23,24,25,26,27,28,29,30,31,32,33]. Twelve trials used only radiation therapy and chemotherapy, and three trials divided patients into two groups (one received chemotherapy and radiotherapy, and the other received radiotherapy alone).

In summary, a total of 378 patients received concurrent S-1 chemotherapy and radiotherapy. It should be noted that the data on tumor response were omitted from 5 studies that did not comply with the RECIST standard.

### 3.2. Effect of Tumor Response Rate

**Figure 2:**
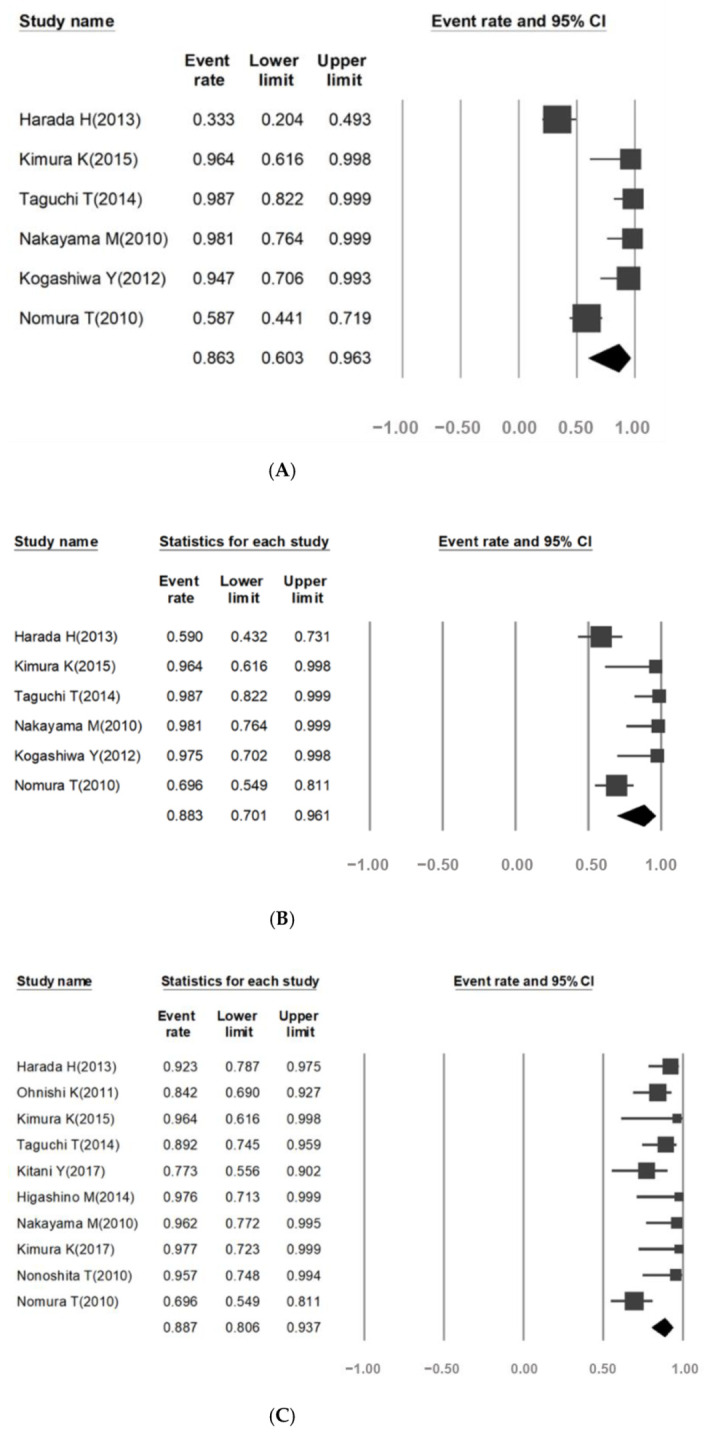
Objective response (**A**), Clinical benefit (**B**) and 3-year local control rate (**C**) of S-1 combined with radiotherapy for Non-nasopharyngeal head and neck cancer. Objective response: Complete Response +Partial Response; Clinical benefit: Complete Response +Partial Response + Stable Disease ≥24 weeks.

This meta-analysis extracted objective response rate (ORR) data from six trials [22,24,25,28,30,33]. The combined ORR calculated using the random effects model was 86.3% and there was high heterogeneity among the studies (95% confidence interval (CI), 60.3–96.3; *I*^2^ = 85.2, *p* < 0.001; Figure 2A).

The subgroup analysis with patients divided by tumor location revealed that the ORR was significantly different between the oral cancer and laryngeal cancer subgroups (46% and 97%, *I*^2^ = 67.3 and 27, respectively, *p* = 0.002, Table 2). The clinical benefit rate (CBR) was estimated using data from six clinical trials [22,24,25,28,30,33]. The overall CBR was 88.3%, and there was high heterogeneity among the studies (95% CI, 70.1–96.1; *I*^2^ = 74.5, *p* = 0.001; Figure 2B). There was a statistically significant difference between the CBR of the oral and laryngeal cancer subgroups (64.0% and 97.8%, *I*^2^ = 77.1 and 18.3, respectively, *p* < 0.001).

### 3.3. Survival Rate

The 3-year local-regional control rate (LCR-3) could be used to analyze 10 clinical trials [22,23,24,25,26,27,28,29,31,33]. The overall LCR-3 rate was 88.7%, and there was high heterogeneity among the studies (95% CI, 80.6–93.7; *I*^2^ = 54.7; *p* = 0.018, Figure 2C). The LCR-3 rate was significantly different between the oral and laryngeal cancer subgroups (82.9% vs. 91.2%, *I*^2^ = 82.63 and 32, respectively, *p* < 0.001).

**Figure 3:**
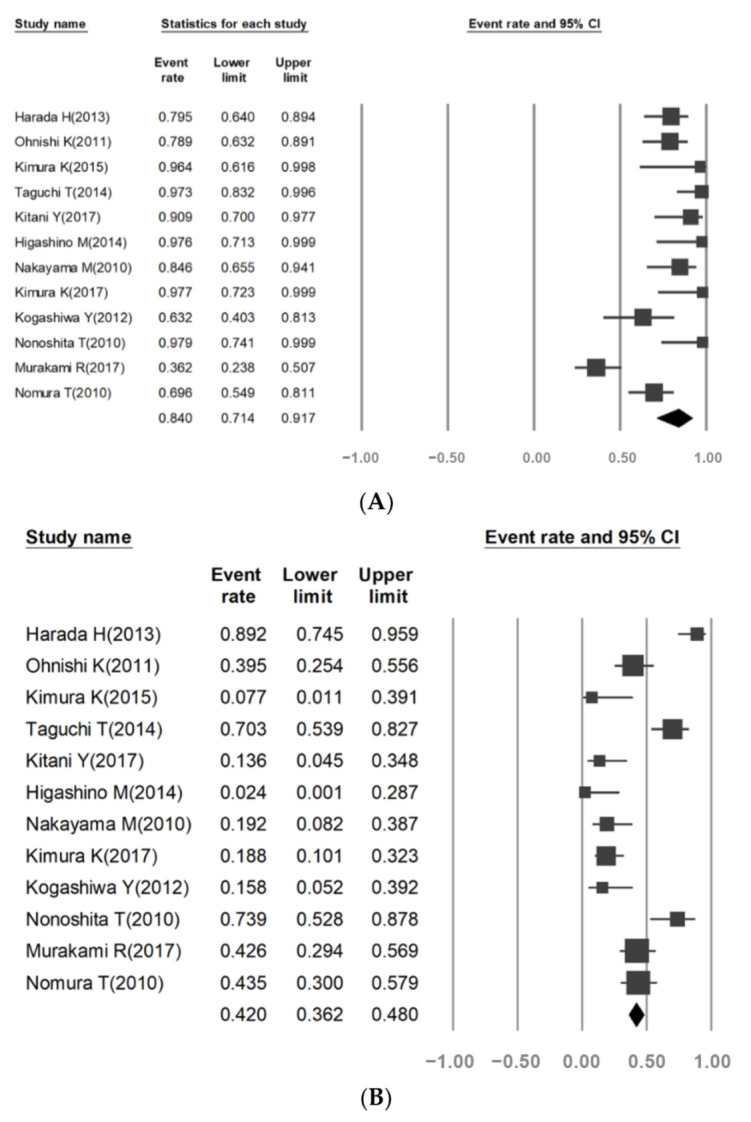
Three-year overall survival (**A**), grade 3/4 adverse events (**B**) of S-1 combined with chemoradiotherapy (CRT) for Non-nasopharyngeal head and neck cancer.

The OS-3 rate was calculated using data from 12 trials [22,23,24,25,26,27,28,29,30,31,32,33]. The overall OS-3 rate was 84.0%, and there was high heterogeneity among the studies (95% CI, 71.4–91.7; *I*^2^ = 79.6, *p* < 0.001; Figure 3A). There was a statistically significant difference in the OS-3 rate between the oral and laryngeal cancer subgroups (60.6% vs. 89.9%, *I*^2^ = 87.76 and 0, respectively, *p* < 0.001).

### 3.4. Grade 3/4 Adverse Events (AEs) Rate

Data on grade 3/4 AEs could be obtained from 12 clinical trials [22,23,24,25,26,27,28,29,30,31,32,33]. The combined rate of grade 3/4 AE occurrence was 42.0%, and there was high heterogeneity among the studies (95%, CI 36.2–48.0; *I*^2^ = 82.85, *p* < 0.001; Figure 3B. There was no significant difference in the rate of grade 3/4 AEs between the oral and laryngeal cancer subgroups (58.2% and 23.8%, respectively, *I*^2^ = 76.4 and 64.33, respectively, *p* = 0.381).

### 3.5. Sensitivity Analysis

Sensitivity analysis was performed by eliminating the single-arm studies, and pooling the remaining OS-3 and LCR-3 data significantly reduced the overall heterogeneity (*I*^2^ = 0 and 36.17). The OS-3 and LCR-3 reached 93.8% (95% CI 0.825–0.980) and 87.0% (0.658–0.959), respectively. (Figure S2) Another sensitivity analysis was conducted via a leave-one-out approach on the OS-3, LCR and grade 3/4 AE data. No clinical trials significantly changed the overall treatment effect (Figure S1).

### 3.6. Publication Bias

**Figure 4:**
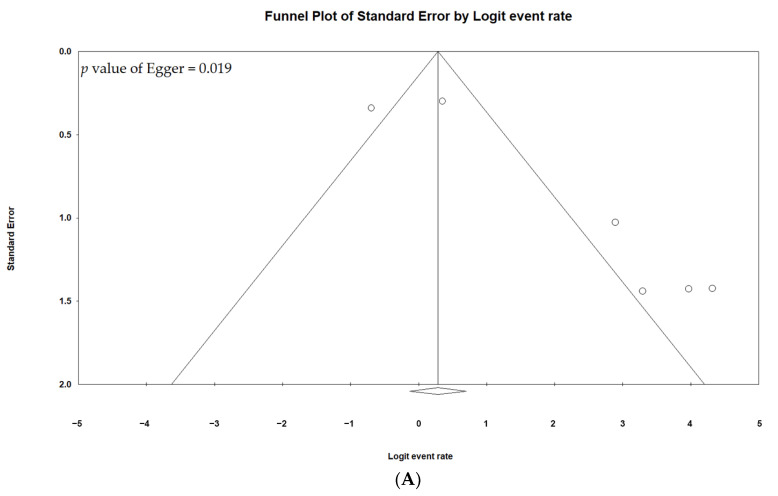

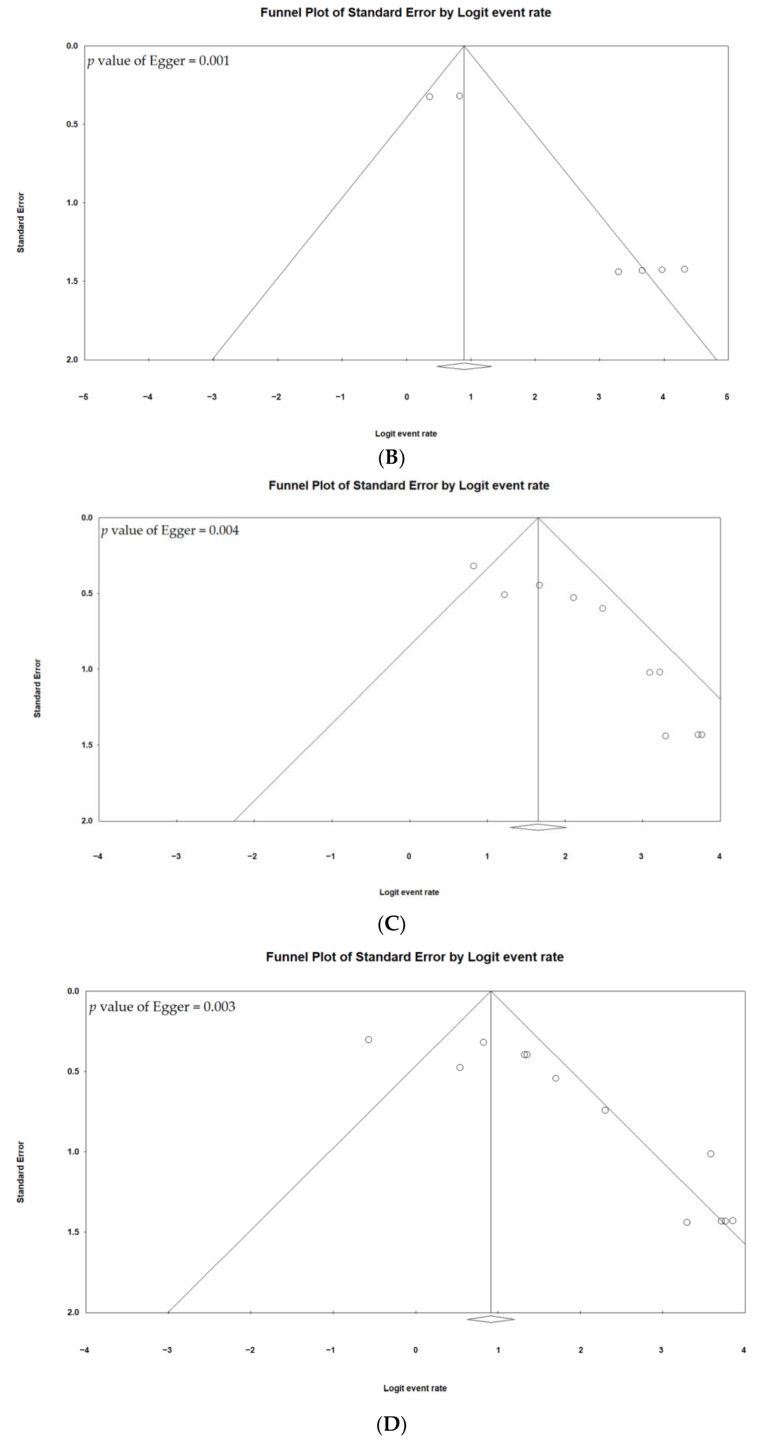

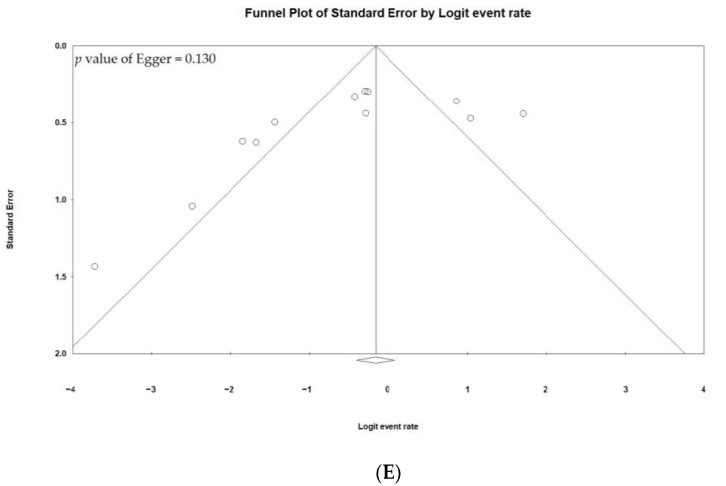
Funnel plots for Objective response (**A**), Clinical benefit (**B**), 3-year local control rate (**C**), 3-year overall survival (**D**), and grade 3/4 adverse events (**E**).

The funnel plots of the LCR-3, OS-3, OR, CB, and grade 3/4 AE data were asymmetrical, as shown in Figure 4, which indicated possible publication bias (Egger’s regression test, *p*  <  0.02), and small study effects were present. The results of the MINORS scale are summarized in Table S2. All 12 studies were observational studies of intermediate quality, three of which had a control group.

## 4. Discussion

Chemotherapy for head and neck squamous cell carcinoma (HNSCC) mainly consists of three drugs: fluorouracil, platinum analogs, and/or taxanes. The efficacy of platinum analogs used as a monochemotherapy was found to be significantly higher than that of other drugs in a meta-analysis of chemotherapy for head and neck cancer (MACH-NC) [34]. Another meta-analysis found that cisplatin used as single agent or in combination with fluorouracil showed the greatest benefit [35]. However, when these drugs are used in large doses, they cause severe toxicity as cisplatin causes mucositis and is nephrotoxic, and fluorouracil is cardiotoxic. Therefore, we selected S-1 as its pharmacokinetic properties are comparable to continuous intravenous infusion of 5-FU [36,37], and the radiosensitive effect of 5-FU is dependent on the length of time the tumor cells are exposed to it [38,39].

A phase II clinical trial in patients with advanced or recurrent HNSCC showed that S-1 treatment achieved clinical antitumor efficacy, with a total remission rate of 28.8% [11]. Some phase I studies of head and neck cancer used S-1 chemotherapy during CCRT [40,41]. The S-1 and CCRT dose schedules were different in each report, and there was no standardization. Therefore, we identified 12 trials with a total of 378 patients who met the strict inclusion criteria for alternative chemoradiation therapy.

The results of this meta-analysis of 12 clinical trials showed that the ORR, CBR and OS rates of non-nasopharyngeal head and neck cancer patients who received S-1 chemoradiotherapy were 86.3% (95% CI 60.3–96.3), 88.3% (95% CI 70.1–96.1) and 84.0% (95% CI 71.4–91.7), respectively. These ratios are higher than those reported in another systematic review, which summarizes the results of Phase II studies. The median ORR, CBR and OS reported in that review were 33.3%, 58% and 78.9%, respectively [22]. The incidence of grade 3/4 AE was 42% (95% CI 36.2–48.0), which seems slightly high. This might be because we considered different types of observed AEs. The 3-year LCR for stage II SCC of the hypopharynx, oropharynx or larynx treated with radiotherapy alone was reported to be 62–80.4% in previous studies [42,43,44,45,46,47]. S-1 chemotherapy was performed simultaneously with conventional radiotherapy in the clinical trials we analyzed for this study, and good results were achieved. The three-year LCR was 88.7% (95% CI, 80.6–93.7%).

Local recurrence in the univariate and multivariate analyses, tumor localization, and classification of T and N were important factors. Although these tumors have common locations and common histologic types, they have distinct characteristics, risk factors, treatment options and prognoses. For example, the consumption of alcohol and tobacco greatly increases the risk for oral squamous cell carcinoma [48]. Oropharyngeal cancer, on the other hand, is strongly related to human papillomavirus (HPV) infection. Treatment planning and oncologic outcome of oropharyngeal cancer is strongly related to HPV infection. While two studies [23,25] enrolled oropharyngeal cancer patients, neither of them was able to clarify their HPV status. This is especially important since all the studies were arranged in Japan. The prevalence of HPV in oropharyngeal cancer is different in Japan than Western countries [49]. Such a condition must be carefully considered before generalizing the data.

We divided the population into two major categories for the subgroup analysis: oral cancer and laryngeal cancer. The comparison of oral and laryngeal cancer showed significant differences in ORR (46% and 97%, respectively, *p* = 0.002), the 3-year OS rate (60.6 and 89.9, respectively, *p* < 0.001) and the median 3-year LCR (82.9% and 91.2%, respectively, *p* < 0.001) The incidence of adverse events was 58.2% in oral cancer patients and 23.8% in laryngeal cancer patients (*p* = 0.381), and there was no significant difference. Most of the patients were in the laryngeal cancer group, which is in line with clinical practice and knowledge that the prognosis of laryngeal cancer is better than the prognosis of HNC at other sites. Part of the reason for this is that the symptoms of laryngeal cancer arise early; thus, many laryngeal cancers are diagnosed early. In addition, the lymphadenopathy of early glottic cancer is much lower than that of other HNSCCs [34].

Our findings are consistent with those from a published meta-analysis that was performed on data from the world’s largest prospective database and showed the overall survival rate, absolute benefit and event-free survival rate of chemotherapy for each tumor site of head and neck cancer [34]. Adjuvant chemotherapy with oral chemotherapeutic agents is recently not the standard of care [50], and has been less studied in Western countries for HNSCC. While S-1 has shown benefit among gastric cancer in China and USA [51,52], we still need further large-scale studies for oral administration of S-1 in HNSCC patients in different countries around the world.

We conducted a sensitivity analysis based on the leave-one-out method to determine the robustness of the relationship between the pooled results and the response rate. Removing the single-arm studies in the sensitivity analysis of the survival rate (OS-3 and LCR-3) significantly reduced the overall heterogeneity. The reason for this might be that the single-arm study survival data were extracted by digitizing software.

This type of meta-analysis has many potential limitations. First, although we excluded studies that lacked important data, not all studies included in the meta-analysis had complete data. Second, the heterogeneity remained a major concern. Possible reasons may include differences in treatment histories, research methods, pathological subtypes and participant numbers among the trials. All analyses were performed using a random effects model to minimize this bias. Third, we had to extract a lot of the survival data by digitizing relevant numbers, which inevitably led to bias. Fourth, because most of the studies were single-arm trials, the scientific quality of findings might be low, and the current results should be interpreted carefully. Finally, as all studies were conducted in Japan, the external validity of the results is limited. In this context, it is difficult to assess the relevance of the potential genetic, environmental, and cultural differences in this study.

## 5. Conclusions

In summary, we conducted a comprehensive evaluation of the use of concurrent S-1 chemotherapy and radiotherapy for the treatment of patients with head and neck cancer patients by a meta-analysis of 12 clinical trials. S-1 chemotherapy combined with CRT for HNSCC patients may result in a good tumor response, high survival rate, and low toxicity. In addition, the prognosis of laryngeal cancer is better than that of other HNSCCs. The results of this meta-analysis show that the risk for severe AEs with the use of S-1 with CRT is acceptable. There is an urgent need to design a reasonable RCT to standardize the concurrent S-1 chemotherapy and radiotherapy treatment plan and confirm the current conclusion.

## Figures and Tables

**Table 1:** Characteristics of the trials including in the meta-analysis.

Author, Year	Country	Trial Design	Schedule	Age (Years) Median (Range)	Patients Evaluated	Tumor Response		LCR-3	OS-3	Grade 3/4 AEs	Evaluation Criteria
						OR	CB				
Harada, 2013 [22]	Japan	Single-arm phase II	CRT with S-1	56.5 (21–75)	39	13	23	91.5	78.9	33	RECIST/NCICTC
Ohnishi, 2011 [23]	Japan	Single-arm Retrospective	CRT with S-1	61 (37–82)	38	NA	NA	83	79	15	NA/CTCAE
Kimura, 2015 [24]	Japan	Single-arm phase I/II	CRT with S-1	67 (59–75)	13	13	13	100	100	1	RECIST/NCICTC
Taguchi, 2014 [25]	Japan	Single-arm phase II	CRT with S-1	68 (49–79)	37	37	37	89	97.2	26	RECIST/CTCAE
Kitani, 2017 [26]	Japan	Retrospective comparative	CRT with S-1	69 (48–91)	22	NA	NA	75	90	3	NA/CTCAE
Higashino, 2014 [27]	Japan	Retrospective comparative	CRT with S-1	66 (53–81)	20	NA	NA	100	100	0	NA/NCITCTC
Nakayama, 2010 [28]	Japan	Single-arm phase I/II	CRT with S-1	66 (53–80)	26	26	26	94.7	85.4	5	RECIST/NCICTC
Kimura, 2017 [29]	Japan	Retrospective comparative	CRT with S-1	68.5 (44–94)	21	NA	NA	100	100	9	NA/CTCAE
Kogashiwa, 2012 [30]	Japan	Single-arm Retrospective	CRT with S-1	84 (75–98)	19	18	19	NA	61.2	3	RECIST/NCICTC
Nonoshita, 2010 [31]	Japan	Single-arm Retrospective	CRT with S-1	64 (52–78)	23	NA	NA	95.4	100	17	NA/CTCAE
Murakami, 2017 [32]	Japan	Single-arm Retrospective	CRT with S-1	79 (45–91)	47	NA	NA	NA	37.3	20	NA/ NCICTC
Nomura, 2010 [33]	Japan	Single-arm phase II	CRT with S-1	76 (50–88)	46	27	32	69	69	20	RECIST/NCICTC

There are some missing data. Objective response: Complete Response + Partial Response; Clinical benefit: Complete Response + Partial Response + Stable Disease ≥ 24 weeks. CRT, chemoradiotherapy; S-1, Tegafur, Gimeracil (CDHP) and potassium oxalate; OR, objective response; CB, clinical benefit; LCR-3, 3-year local control rate; OS-3, 3-year overall survival; AEs, adverse events; RECIST, Response Evaluation Criteria in Solid Tumors; NCICTC, National Cancer Institute Common Toxicity Criteria; CTCAE, Common Terminology Criteria for Adverse Events; NA, not available.

**Table 2:** Subgroup analysis of standardized mean differences based on primary sites.

Endpoints and Adverse Events	Oral Cancer		Laryngeal Cancer		*p* Value
	No. of Trials	Rate % (95%CI)	No. of Trials	Rate % (95%CI)	
Objective response	2	46.0 (23.5–70.4)	3	97.0 (90.2–99.1)	0.002
Clinical benefit	2	64.0 (53.8–74.0)	3	97.8 (82.2–99.9)	<0.001
3-year local control rate *	2	82.9 (49–96.1)	7	91.2 (83.5–95.5)	<0.001
3-year overall survival *	3	60.6 (35.5–81.1)	7	89.9 (78.4–95.6)	<0.001
Grade 3/4 adverse events	3	58.2 (31.8–80.6)	6	23.8 (8.8–50.5)	0.381

Objective response: Complete Response + Partial Response; Clinical benefit: Complete Response + Partial Response + Stable Disease ≥ 24 weeks. * The heterogeneity of 3-year local control rate and Three-year overall survival in laryngeal cancer was significantly lower than that in oral cancer.

## Data Availability

Data generated are included within the manuscript and supplementary materials.

## Supplementary Materials

The following are available online at https://www.mdpi.com/article/10.3390/cancers13122971/s1, Figure S1. Sensitivity analysis on OR (A), CB (B), LCR-3 (C), OS-3(D), and grade 3/4 AEs (E) rates, Figure S2. Sensitivity analysis of excluding study design as non–comparative study: incidence of LCR-3 (A), OS-3 (B), and grade 3/4 AEs (C), Table S1: Preferred Reporting Items for Systematic Reviews and Meta-Analyses (PRISMA) Checklist, Table S2: Quality Assessments.
