# Supplementary material for: The Efficacy and Adverse Events in Patients with Head and Neck Cancer Following Radiotherapy Combined with S-1 Therapy: A Meta-Analysis
Source: Cancers (Basel). 2021 Jun 13;13(12):2971. doi: 10.3390/cancers13122971 (PMC8231857; doi:10.3390/cancers13122971)
Supplement: Supplementary file 1 [file cancers-13-02971-s001.zip › cancers-1225926-supplementary.pdf]

Supplementary Information: The Efficacy and Adverse Events in Patients with Head and Neck Cancer Following Radiotherapy Combined with S-1 Therapy: A Meta-Analysis

Hung-Sheng Shih, Hong-Jie Jhou, Yang-Hao Ou, Yen-Tze Liu, Chew-Teng Kor, Andy Wei-Ge Chen, Mu-Kuan Chen

Contents

Supplementary Information 1. Search strategy
Supplementary Information 2. Preferred Reporting Items for Systematic Reviews and Meta-Analyses (PRISMA) Checklist
Supplementary Information 3. Assessment of risk of bias
Supplementary Information 4. Reference list of full-text screening studies
Supplementary Information 5. Sensitivity analysis

Information 1: Search Strategy

Embase

#1 head and neck tumor:ti, ab
#2 head and neck cancer*:ti, ab
#3 neck neoplasm*:ti, ab
#4 #1 OR #2 OR #3
#5 S-1:ti, ab
#6 gimeracil plus oteracil potassium plus tegafur*:ti, ab
#7 #5 OR #6
#8 #1 AND #5
#9 #2 AND #6
#10 #8 OR #9

Cochrane Library

#1 [head and neck neoplasms] OR [head and neck cancer]
#2 (“S-1”)
#3 [mh " gimeracil plus oteracil potassium plus tegafur "]
#4 #2 or #3
#5 #1 or #4

PubMed

#1 head and neck cancer
#2 head and neck tumor*
#3 head and neck neoplasms*
#4 S-1
#5 gimeracil plus oteracil potassium plus tegafur
#6 (#1OR#2OR#3)AND (#4 OR #5)

Information 2. Table S1. Preferred Reporting Items for Systematic Reviews and Meta-Analyses (PRISMA) checklist.

Section and Topic	Item #	Checklist item	Location where item is reported
**TITLE**			
Title	1	Identify the report as a systematic review.	1
**ABSTRACT**			
Abstract	2	See the PRISMA 2020 for Abstracts checklist.	1
**INTRODUCTION**			
Rationale	3	Describe the rationale for the review in the context of existing knowledge.	2
Objectives	4	Provide an explicit statement of the objective(s) or question(s) the review addresses.	2
**METHODS**			
Eligibility criteria	5	Specify the inclusion and exclusion criteria for the review and how studies were grouped for the syntheses.	3
Information sources	6	Specify all databases, registers, websites, organisations, reference lists and other sources searched or consulted to identify studies. Specify the date when each source was last searched or consulted.	2-3
Search strategy	7	Present the full search strategies for all databases, registers and websites, including any filters and limits used.	Appendices
Selection process	8	Specify the methods used to decide whether a study met the inclusion criteria of the review, including how many reviewers screened each record and each report retrieved, whether they worked independently, and if applicable, details of automation tools used in the process.	2-3
Data collection process	9	Specify the methods used to collect data from reports, including how many reviewers collected data from each report, whether they worked independently, any processes for obtaining or confirming data from study investigators, and if applicable, details of automation tools used in the process.	3-4
Data items	10a	List and define all outcomes for which data were sought. Specify whether all results that were compatible with each outcome domain in each study were sought (e.g., for all measures, time points, analyses) and, if not, the methods used to decide which results to collect.	3
	10b	List and define all other variables for which data were sought (e.g., participant and intervention characteristics, funding sources). Describe any assumptions made about any missing or unclear information.	3
Study risk of bias assessment	11	Specify the methods used to assess risk of bias in the included studies, including details of the tool(s) used, how many reviewers assessed each study and whether they worked independently, and if applicable, details of automation tools used in the process.	3-4
Effect measures	12	Specify for each outcome the effect measure(s) (e.g., risk ratio, mean difference) used in the synthesis or presentation of results.	3
Synthesis methods	13a	Describe the processes used to decide which studies were eligible for each synthesis (e.g., tabulating the study intervention characteristics and comparing against the planned groups for each synthesis (item #5)).	3
	13b	Describe any methods required to prepare the data for presentation or synthesis, such as handling of missing summary statistics, or data conversions.	3
	13c	Describe any methods used to tabulate or visually display results of individual studies and syntheses.	3
	13d	Describe any methods used to synthesize results and provide a rationale for the choice(s). If meta-analysis was performed, describe the model(s), method(s) to identify the presence and extent of statistical heterogeneity, and software package(s) used.	3
	13e	Describe any methods used to explore possible causes of heterogeneity among study results (e.g., subgroup analysis, meta-regression).	3
	13f	Describe any sensitivity analyses conducted to assess robustness of the synthesized results.	3

Section and Topic	Item #	Checklist item	Location where item is reported
Reporting bias assessment	14	Describe any methods used to assess risk of bias due to missing results in a synthesis (arising from reporting biases).	3-4
Certainty assessment	15	Describe any methods used to assess certainty (or confidence) in the body of evidence for an outcome.	4
**RESULTS**			
Study selection	16a	Describe the results of the search and selection process, from the number of records identified in the search to the number of studies included in the review, ideally using a flow diagram.	4
	16b	Cite studies that might appear to meet the inclusion criteria, but which were excluded, and explain why they were excluded.	4
Study characteristics	17	Cite each included study and present its characteristics.	4, Table 1
Risk of bias in studies	18	Present assessments of risk of bias for each included study.	Appendices
Results of individual studies	19	For all outcomes, present, for each study: (a) summary statistics for each group (where appropriate) and (b) an effect estimate and its precision (e.g., confidence/credible interval), ideally using structured tables or plots.	9-11
Results of syntheses	20a	For each synthesis, briefly summarise the characteristics and risk of bias among contributing studies.	9-11
	20b	Present results of all statistical syntheses conducted. If meta-analysis was done, present for each the summary estimate and its precision (e.g., confidence/credible interval) and measures of statistical heterogeneity. If comparing groups, describe the direction of the effect.	9-11
	20c	Present results of all investigations of possible causes of heterogeneity among study results.	9-11
	20d	Present results of all sensitivity analyses conducted to assess the robustness of the synthesized results.	9-11
Reporting biases	21	Present assessments of risk of bias due to missing results (arising from reporting biases) for each synthesis assessed.	11
Certainty of evidence	22	Present assessments of certainty (or confidence) in the body of evidence for each outcome assessed.	Appendices
**DISCUSSION**			
Discussion	23a	Provide a general interpretation of the results in the context of other evidence.	13
	23b	Discuss any limitations of the evidence included in the review.	14
	23c	Discuss any limitations of the review processes used.	14
	23d	Discuss implications of the results for practice, policy, and future research.	14
**OTHER INFORMATION**			
Registration and protocol	24a	Provide registration information for the review, including register name and registration number, or state that the review was not registered.	2
	24b	Indicate where the review protocol can be accessed, or state that a protocol was not prepared.	13
	24c	Describe and explain any amendments to information provided at registration or in the protocol.	13
Support	25	Describe sources of financial or non-financial support for the review, and the role of the funders or sponsors in the review.	13
Competing interests	26	Declare any competing interests of review authors.	13
Availability of data, code and other materials	27	Report which of the following are publicly available and where they can be found: template data collection forms; data extracted from included studies; data used for all analyses; analytic code; any other materials used in the review.	13

Information 3 Table S2. Quality Assessments

Newcastle-Ottawa Scale quality assessment scale for included studies								
**First author, year**	**Representativeness of the S-1 with chemoradiotherapy**	**Selection of control**	**Ascertainment of S-1 with chemoradio-therapy**	**Demonstration that out-come of interest was not present at start of study**	**Comparability of co-horts on the basis of the design or analysis**	**Assessment of outcome**	**Was follow-up long enough for out-comes to occur**	**Adequacy of follow up**
Harada et al 2013	❋	–	❋	❋	–	❋	❋	❋
Ohnishi et al 2011	❋	–	❋	❋	–	❋	❋	❋
Kimura et al 2015	❋	–	❋	❋	–	❋	❋	❋
Taguchi et al 2014	❋	–	❋	❋	–	❋	❋	❋
Kitani et al 2017	❋	❋❋	❋	❋	❋❋	❋	❋	❋
Higashino et al 2014	❋	❋❋	❋	❋	❋❋	❋	❋	❋
Nakayama et al 2010	❋	–	❋	❋	–	❋	❋	❋
Kimura et al 2017	❋	❋❋	❋	❋	❋❋	❋	❋	❋
Kogashiwa et al 2012	❋	–	❋	❋	–	❋	–	❋
Nonoshita et al 2010	❋	–	❋	❋	–	❋	❋	❋
Murakami et al 2017	❋	–	❋	❋	–	❋	–	❋
Nomura et al 2010	❋	–	❋	❋	–	❋	❋	❋

The revised and validated version of Methodological Index for Non-randomized Studies (MINORS)													
									**Additional criteria in the case of comparative study**				
**First author, year**	**A clearly stated aim**	**Inclusion of consecutive patients**	**Prospective collection of data**	**Endpoints appropriate to the aim of the study**	**Unbiased assessment of the study endpoint**	**Follow-up period appropriate to the aim of the study**	**Loss to follow up less than 5%**	**Prospective calculation of the study size**	**An adequate control group**	**Contemporary groups**	**Baseline equivalence of groups**	**Adequate statistical analyses**	**score**
Harada et al 2013	2	2	0	2	1	1	2	1	NA	NA	NA	NA	11
Ohnishi et al 2011	2	2	0	2	0	1	2	1	NA	NA	NA	NA	10
Kimura et al 2015	2	2	2	2	1	1	1	0	NA	NA	NA	NA	11
Taguchi et al 2014	2	2	2	2	1	2	1	1	NA	NA	NA	NA	13
Kitani et al 2017	2	2	0	2	1	2	1	1	2	2	2	2	19
Higashino et al 2014	2	2	0	2	1	1	1	1	2	1	2	2	17
Nakayama et al 2010	2	2	2	2	2	2	2	1	NA	NA	NA	NA	15
Kimura et al 2017	2	2	0	2	1	1	1	1	2	2	1	2	17
Kogashiwa et al 2012	2	2	2	1	1	1	2	1	NA	NA	NA	NA	12
Nonoshita et al 2010	2	2	0	2	2	2	2	1	NA	NA	NA	NA	13
Murakami et al 2017	2	2	0	2	2	2	0	1	NA	NA	NA	NA	11
Nomura et al 2010	2	2	2	2	2	2	2	1	NA	NA	NA	NA	15

* The items are scored 0 (not reported), 1 (reported but inadequate) or 2 (reported and adequate). The global ideal score being 16 for non-comparative studies and 24 for comparative.

Information 4 Reference list of full-text screening studies

Included studies (No.22 to No.33)

1. Cancer statistics. JAMA **2013**, *310*, 982, Doi: 10.1001/jama.2013.5289
2. Montagna, E.; Cancello, G.; Dellapasqua, S.; Munzone, E.; Colleoni, M. Metronomic therapy and breast cancer: A systematic review. *Cancer Treat. Rev.* **2014**, *40*, 942–950, doi:10.1016/j.ctrv.2014.06.002.
3. A Salem, D.; Gado, N.M.; Abdelaziz, N.N.; E Essa, A.; Abdelhafeez, Z.M.; Kamel, T.H. Phase II trial of metronomic chemotherapy as salvage therapy for patients with metastatic breast cancer. *J. Egypt. Natl. Cancer Inst.* **2008**, *20*, 134–40.
4. Tatsumi, K.; Fukushima, M.; Shirasaka, T.; Fujii, S. Inhibitory effects of pyrimidine, barbituric acid and pyridine derivatives on 5-fluorouracil degradation in rat liver extracts. *Jpn. J. Cancer Res.* 1987, *78*, 748–55.
5. Diasio, R.B. Clinical implications of dihydropyrimidine dehydrogenase inhibition. *Oncol.* **1999**, *13*, 17–21.
6. Shirasaka, T.; Shimamato, Y.; Ohshimo, H.; Yamaguchi, M.; Kato, T.; Yonekura, K.; Fukushima, M. Development of a novel form of an oral 5-fluorouracil derivative (S-1) directed to the potentiation of the tumor selective cytotoxicity of 5-fluorouracil by two biochemical modulators. *Anti-Cancer Drugs* **1996**, *7*, 548–557, doi:10.1097/00001813-199607000-00010.
7. Al-Sarraf, M.; Leblanc, M.; Giri, P.G.; Fu, K.K.; Cooper, J.; Vuong, T.; A Forastiere, A.; Adams, G.; A Sakr, W.; E Schuller, D.; et al. Chemoradiotherapy versus radiotherapy in patients with advanced nasopharyngeal cancer: phase III randomized Intergroup study 0099. *J. Clin. Oncol.* **1998**, *16*, 1310–1317, doi:10.1200/jco.1998.16.4.1310.
8. Lin, J.-C.; Jan, J.-S.; Hsu, C.-Y.; Liang, W.-M.; Jiang, R.-S.; Wang, W.-Y. Phase III Study of Concurrent Chemoradiotherapy Versus Radiotherapy Alone for Advanced Nasopharyngeal Carcinoma: Positive Effect on Overall and Progression-Free Survival. *J. Clin. Oncol.* **2003**, *21*, 631–637, doi:10.1200/jco.2003.06.158.
9. Chan, A.T.C.; Teo, P.M.L.; Ngan, R.; Leung, T.W.; Lau, W.; Zee, B.C.-Y.; Leung, S.; Cheung, F.; Yeo, W.; Yiu, H.; et al. Concurrent Chemotherapy-Radiotherapy Compared With Radiotherapy Alone in Locoregionally Advanced Nasopharyngeal Carcinoma: Progression-Free Survival Analysis of a Phase III Randomized Trial. *J. Clin. Oncol.* **2002**, *20*, 2038–2044, doi:10.1200/jco.2002.08.149.
10. Chan, A.T.C.; Teo, P.M.; Leung, T.W.; Leung, S.F.; Lee, W.Y.; Yeo, W.; Choi, P.H.; Johnson, P.J. A prospective randomized study of chemotherapy adjunctive to definitive radiotherapy in advanced nasopharyngeal carcinoma. *Int. J. Radiat. Oncol.* **1995**, *33*, 569–577, doi:10.1016/0360-3016(95)00218-n.
11. Rossi, A.; Molinari, R.; Boracchi, P.; Del Vecchio, M.; Marubini, E.; Nava, M.; Morandi, L.; Zucali, R.; Pilotti, S.; Grandi, C. Adjuvant chemotherapy with vincristine, cyclophosphamide, and doxorubicin after radiotherapy in local-regional nasopharyngeal cancer: results of a 4-year multicenter randomized study. *J. Clin. Oncol.* **1988**, *6*, 1401–1410, doi:10.1200/jco.1988.6.9.1401.
12. Inuyama, Y.; Kida, A.; Tsukuda, M.; Kohno, N.; Satake, B. [Late phase II study of S-1 in patients with advanced head and neck cancer]. *Gan kagaku ryoho. Cancer Chemother.* **2001**, *28*, 1381–90.
13. Inuyama, Y.; Kida, A.; Tsukuda, M.; Kohno, N.; Satake, B. [Early phase II study of S-1 in patients with advanced head and neck cancer. S-1 Cooperative Study Group (Head and Neck Working Group)]. *Gan kagaku ryoho. Cancer Chemother.* **1998**, *25*, 1151–8.
14. Hutton, B.; Salanti, G.; Caldwell, D.M.; Chaimani, A.; Schmid, C.; Cameron, C.; Ioannidis, J.P.; E Straus, S.; Thorlund, K.; Jansen, J.P.; et al. The PRISMA Extension Statement for Reporting of Systematic Reviews Incorporating Network Meta-analyses of Health Care Interventions: Checklist and Explanations. *Ann. Intern. Med.* **2015**, *162*, 777–784, doi:10.7326/m14-2385.
15. Furlan, A.D.; Pennick, V.; Bombardier, C.; Van Tulder, M. 2009 Updated Method Guidelines for Systematic Reviews in the Cochrane Back Review Group. *Spine* **2009**, *34*, 1929–1941, doi:10.1097/brs.0b013e3181b1c99f.
16. Roberts, C.; Torgerson, D. Understanding controlled trials: Baseline imbalance in randomised controlled trials. *BMJ* **1999**, *319*, 185, doi:10.1136/bmj.319.7203.185.
17. Egger, M.; Smith, G.D.; Schneider, M.; Minder, C. Bias in meta-analysis detected by a simple, graphical test. *BMJ* **1997**, *315*, 629, doi:10.1136/bmj.315.7109.629.
18. Begg, C.B.; Mazumdar, M. Operating Characteristics of a Rank Correlation Test for Publication Bias. *Biometrics* **1994**, *50*, 1088, doi:10.2307/2533446.
19. Higgins, J.P.T.; Thomas, J.; Chandler, J.; Cumpston, M.; Li, T.; Page, M.J.; Welch VA. Eds. Cochrane Handbook for Systematic Reviews of Interventions. 2nd ed.; John Wiley & Sons: Chichester, UK, 2019.
20. Higgins, J.P.T.; Thompson, S.G.; Deeks, J.J.; Altman, D.G. Measuring inconsistency in meta-analyses. *BMJ* **2003**, *327*, 557–560, doi:10.1136/bmj.327.7414.557.
21. Guyatt, G.; Oxman, A.D.; Akl, E.A.; Kunz, R.; Vist, G.; Brozek, J.; Norris, S.; Falck-Ytter, Y.; Glasziou, P.; Debeer, H. GRADE guidelines: 1. Introduction—GRADE evidence profiles and summary of findings tables. *J. Clin. Epidemiology* **2011**, *64*, 383–394, doi:10.1016/j.jclinepi.2010.04.026.
22. Harada, H.; Omura, K.; Tomioka, H.; Nakayama, H.; Hiraki, A.; Shinohara, M.; Yoshihama, Y.; Shintani, S. Multicenter phase II trial of preoperative chemoradiotherapy with S-1 for locally advanced oral squamous cell carcinoma. *Cancer Chemother. Pharmacol.* **2013**, *71*, 1059–1064, doi:10.1007/s00280-013-2101-5.
23. Ohnishi, K.; Shioyama, Y.; Nakamura, K.; Nakashima, T.; Ohga, S.; Nonoshita, T.; Yoshitake, T.; Terashima, K.; Komune, S.; Honda, H. Concurrent Chemoradiotherapy with S-1 as First-line Treatment for Patients with Oropharyngeal Cancer. *J. Radiat. Res.* **2011**, *52*, 47–53, doi:10.1269/jrr.10081.
24. Kimura, K.; Itoh, Y.; Okada, T.; Nakahara, R.; Kawamura, M.; Kubota, S.; Itoh, J.; Hiramatsu, M.; Fujimoto, Y.; Shibata, T.; et al. Critical evaluation of a prospective study of concurrent chemoradiotherapy with S-1 for early glottic carcinoma. *Anticancer. Res.* **2015**, *35*, 2385–90.

25. Taguchi, T.; Takahashi, M.; Nishimura, G.; Shiono, O.; Komatsu, M.; Sano, D.; Sakuma, Y.; Tanigaki, Y.; Kubota, A.; Taguri, M.; et al. Phase II Study of Concurrent Chemoradiotherapy with S-1 in Patients with Stage II (T2N0M0) Squamous Cell Carcinoma of the Pharynx or Larynx. *Jpn. J. Clin. Oncol.* **2014**, *44*, 1158–1163, doi:10.1093/jjco/hyu154.
26. Kitani, Y., Kubota, A.; Furukawa, M; Hori, Y.; Nakayama, Y.; Nonaka, T.; Mizoguchi, N.; Yuka Kitani, Y.; Hatakeyama, H.; Oridate, O. Impact of combined modality treatment with radiotherapy and S-1 on T2N0 laryngeal cancer: Possible improvement in survival through the prevention of second primary cancer and distant metastasis. *Oral Oncol.* **2017**, *71*, 54–59.
27. Higashino, M.; Kawata, R.; Lee, K.; Nishikawa, S.; Ichihara, S.; Uesugi, Y. Radiotherapy concurrent with S-1 and radiotherapy alone for T2N0 glottic carcinoma: A retrospective comparative study. *Auris Nasus Larynx* **2014**, *41*, 364–368, doi:10.1016/j.anl.2014.02.001.
28. Nakayama, M.; Hayakawa, K.; Okamoto, M.; Niibe, Y.; Ishiyama, H.; Kotani, S. Phase I/II Trial of Concurrent Use of S-1 and Radiation Therapy for T2 Glottic Cancer. *Jpn. J. Clin. Oncol.* **2010**, *40*, 921–926, doi:10.1093/jjco/hyq077.
29. Kimura, K.; Itoh, Y.; Okada, T.; Kubota, S.; Kawamura, M.; Nakahara, R.; Oie, Y.; Kozai, Y.; Takase, Y.; Tsuzuki, H.; et al. Optimized treatment strategy of radiotherapy for early glottic squamous cell carcinomas: An initial analysis. *Nagoya J. Med Sci. 79*, 331–338.
30. Kogashiwa, Y.; Nagafuji, H.; Kohno, N. Feasibility of concurrent chemoradiotherapy with S-1 administered on alternate days for elderly patients with head and neck cancer. *Anticancer. Res.* **2012**, *32*, 4035–40.
31. Nonoshita, T.; Shioyama, Y.; Nakamura, K.; Nakashima, T.; Ohga, S.; Yoshitake, T.; Ohnishi, K.; Terashima, K.; Asai, K.; Honda, H. Concurrent Chemoradiotherapy with S-1 for T2N0 Glottic Squamous Cell Carcinoma. *J. Radiat. Res.* **2010**, *51*, 481–484, doi:10.1269/jrr.09134.
32. Murakami, R.; Semba, A.; Kawahara, K.; Matsuyama, K.; Hiraki, A.; Nagata, M.; Toya, R.; Yamashita, Y.; Oya, N.; Nakayama, H. Concurrent chemoradiotherapy with S-1 in patients with stage III–IV oral squamous cell carcinoma: A retrospective analysis of nodal classification based on the neck node level. *Mol. Clin. Oncol.* **2017**, *7*, 140–144, doi:10.3892/mco.2017.1276.
33. Nomura, T.; Murakami, R.; Toya, R.; Teshima, K.; Nakahara, A.; Hirai, T.; Hiraki, A.; Nakayama, H.; Yoshitake, Y.; Ota, K.; et al. Phase II Study of Preoperative Concurrent Chemoradiation Therapy With S-1 in Patients With T4 Oral Squamous Cell Carcinoma. *Int. J. Radiat. Oncol.* **2010**, *76*, 1347–1352, doi:10.1016/j.ijrobp.2009.03.055.
34. Pignon, J.-P.; le Maître, A.; Maillard, E.; Bourhis, J. Meta-analysis of chemotherapy in head and neck cancer (MACH-NC): An update on 93 randomised trials and 17,346 patients. *Radiother. Oncol.* **2009**, *92*, 4–14, doi:10.1016/j.radonc.2009.04.014.
35. Budach, W.; Hehr, T.; Budach, V.; Belka, C.; Dietz, K. A meta-analysis of hyperfractionated and accelerated radiotherapy and combined chemotherapy and radiotherapy regimens in unresected locally advanced squamous cell carcinoma of the head and neck. *BMC Cancer* **2006**, *6*, 28, doi:10.1186/1471-2407-6-28.
36. Yamada, Y. [Plasma concentrations of 5-fluorouracil and F-beta-alanine following oral administration of S-1, a dihydropyrimidine dehydrogenase inhibitory fluoropyrimidine, as compared with protracted venous infusion of 5-fluorouracil]. *Gan kagaku ryoho. Cancer Chemother.* **2006**, *33*, 816–20.
37. Hirata, K.; Horikoshi, N.; Aiba, K.; Okazaki, M.; Denno, R.; Sasaki, K.; Nakano, Y.; Ishizuka, H.; Yamada, Y.; Uno, S.; et al. Pharmacokinetic study of S-1, a novel oral fluorouracil antitumor drug. *Clin. Cancer Res.* **1999**, *5*, 2000–5.
38. Byfield, J.E.; Calabro-Jones, P.; Klisak, I.; Kulhanian, F. Pharmacologic requirements for obtaining sensitization of human tumor cells in vitro to combined 5-fluorouracil or ftorafur and X rays. *Int. J. Radiat. Oncol.* **1982**, *8*, 1923–1933, doi:10.1016/0360-3016(82)90451-5.
39. Smalley, S.R.; Kimler, B.F.; Evans, R.G. 5-Fluorouracil modulation of radiosensitivity in cultured human carcinoma cells. *Int. J. Radiat. Oncol.* **1991**, *20*, 207–211, doi:10.1016/0360-3016(91)90091-h.
40. Nakata, K.; Sakata, K.-I.; Someya, M.; Miura, K.; Hayashi, J.; Hori, M.; Takagi, M.; Himi, T.; Kondo, A.; Hareyama, M. Phase I study of oral S-1 and concurrent radiotherapy in patients with head and neck cancer. *J. Radiat. Res.* **2013**, *54*, 679–683, doi:10.1093/jrr/rrs133.
41. Fujimoto, Y.; Kato, S.; Itoh, Y.; Naganawa, S.; Nakashima, T. A phase I study of concurrent chemoradiotherapy using oral s-1 for head and neck cancer. *Anticancer. Res.* **2014**, *34*, 209–13.
42. Inoue, T., Inoue, T.; Ikeda, H.; Teshima, T.; Murayama, S. Prognostic factor of telecobalt therapy for early glottic carcinoma. *Cancer* **1992,** *70*, 2797–2801.
43. Mendenhall, W.M.; Werning, J.W.; Hinerman, R.W.; Amdur, R.J.; Villaret, D.B. Management of T1-T2 glottic carcinomas. *Cancer* **2004**, *100*, 1786–1792, doi:10.1002/cncr.20181.
44. Inoue, T.; Matayoshi, Y.; Inoue, T.; Ikeda, H.; Teshima, T.; Murayama, S. Prognostic factors in telecobalt therapy for early supraglottic carcinoma. *Cancer* **1993,** *72*, 57–61.
45. Wong, C.S.; Ang, K.K.; Fletcher, G.H.; Thames, H.D.; Peters, L.J.; Byers, R.M.; Oswald, M.J. Definitive radiotherapy for squamous cell carcinoma of the tonsillar fossa. *Int. J. Radiat. Oncol.* **1989**, *16*, 657–662, doi:10.1016/0360-3016(89)90481-1.
46. Mendenhall, W.M.; Parsons, J.T.; Stringer, S.P.; Cassisi, N.J.; Million, R.R. Radiotherapy alone or combined with neck dissection for T1−T2 carcinoma of the pyriform sinus: An alternative to conservation surgery. *Int. J. Radiat. Oncol.* **1993**, *27*, 1017–1027, doi:10.1016/0360-3016(93)90518-z.
47. Selek, U.; Garden, A.S.; Morrison, W.H.; El-Naggar, A.K.; Rosenthal, D.; Ang, K. Radiation therapy for early-stage carcinoma of the oropharynx. *Int. J. Radiat. Oncol.* **2004**, *59*, 743–751, doi:10.1016/j.ijrobp.2003.12.002.
48. Mello, F.W.; Melo, G.; Pasetto, J.J.; Silva, C.A.B.; Warnakulasuriya, S.; Rivero, E.R.C. The synergistic effect of tobacco and alcohol consumption on oral squamous cell carcinoma: a systematic review and meta-analysis. *Clin. Oral Investig.* **2019**, *23*, 2849–2859, doi:10.1007/s00784-019-02958-1.

Information 5. Figure S1. Sensitivity analysis on OR (A), CB (B), LCR-3 (C), OS-3(D), and grade 3/4 AEs (E) rates.

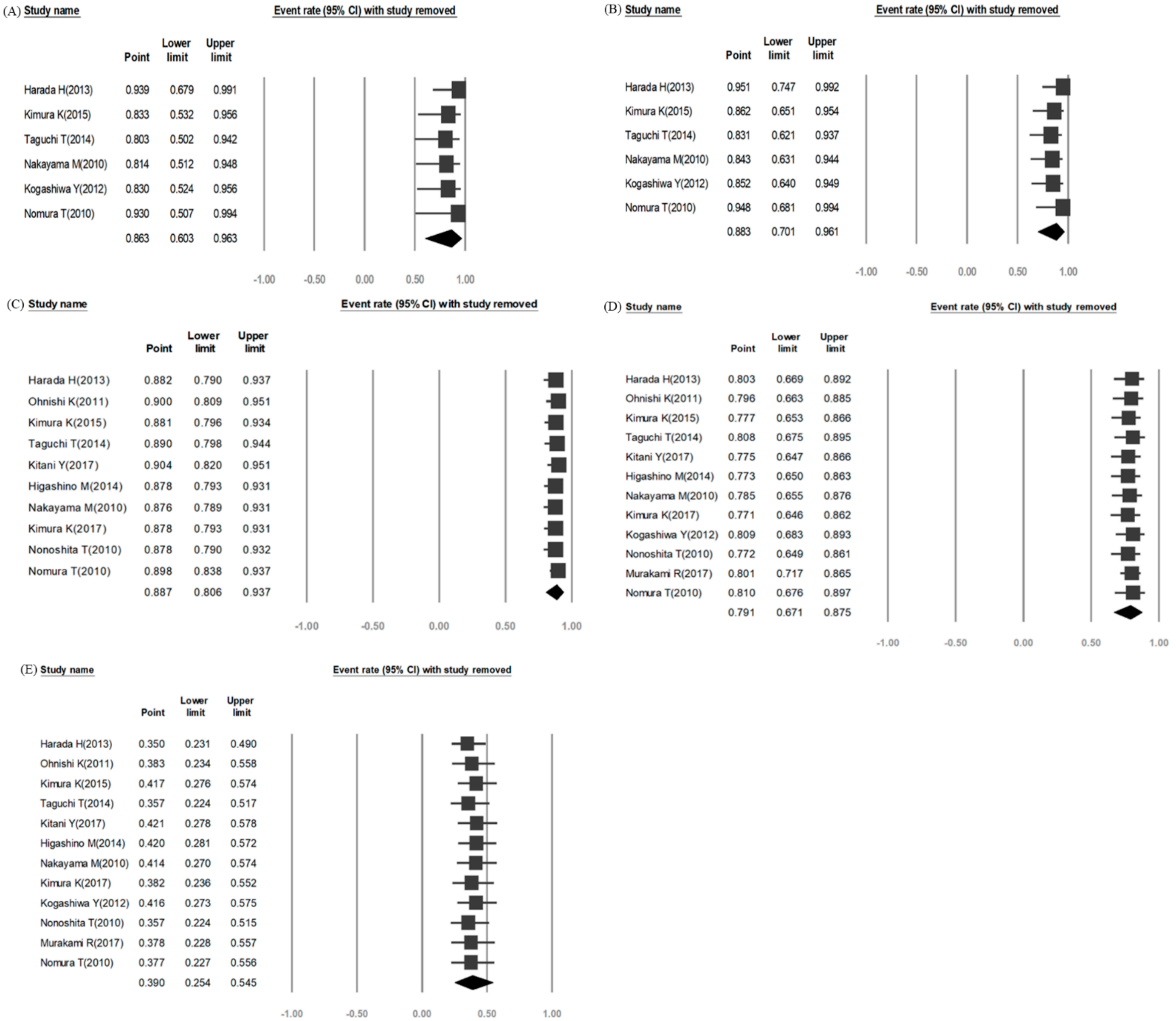

* OR, objective response; CB, clinical benefit; LCR-3, 3-year local control rate; OS-3, 3-year overall survival; AEs, adverse events

Figure S2. Sensitivity analysis of excluding study design as non–comparative study: incidence of LCR-3 (A), OS-3 (B), and grade 3/4 AEs (C)**.**

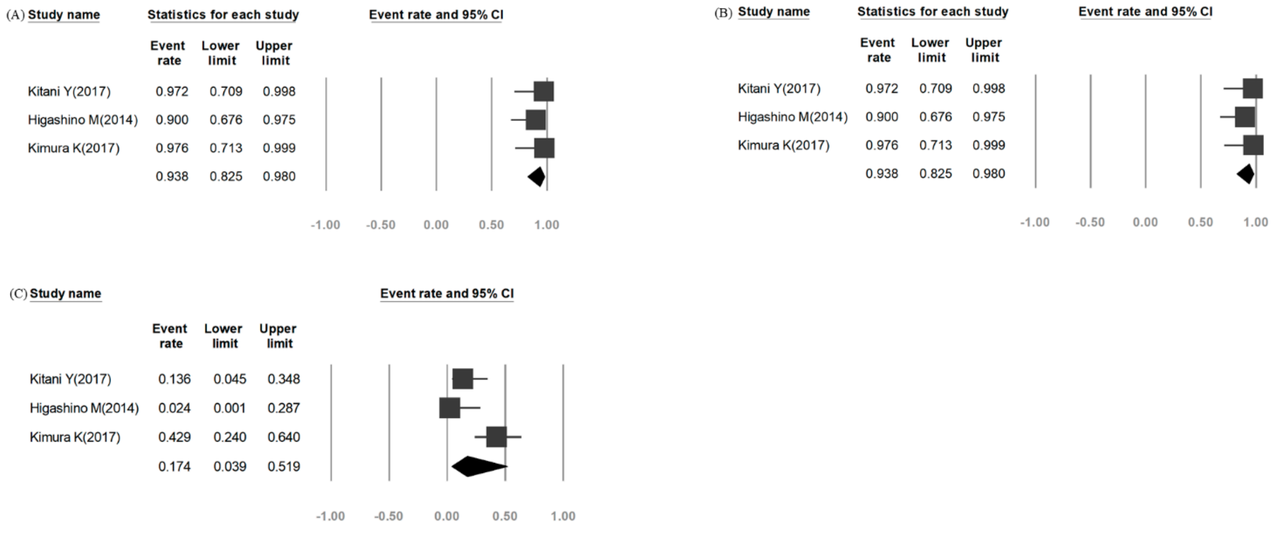

* LCR-3, 3-year local control rate; OS-3, 3-year overall survival; AEs, adverse events
